# Thermal Energy Storage Using Hybrid Nanofluid Phase Change Material (PCM) Based on Waste Sludge Incorp Rated ZnO/α-Fe_2_O_3_

**DOI:** 10.3390/nano14070604

**Published:** 2024-03-28

**Authors:** Ehssan Ahmed Hassan, Maha A. Tony, Mohamed M. Awad

**Affiliations:** 1 Department of Biology, College of Science and Humanities, Prince Sattam bin Abdulaziz University, Alkharj 11942, Saudi Arabia; 2 Department of Zoology, Faculty of Science, Suez Canal University, Ismailia 8366004, Egypt; 3 Basic Engineering Science Department, Faculty of Engineering, Menoufia University, Shebin El-Kom 6131567, Egypt; 4 Advanced Materials/Solar Energy and Environmental Sustainability (AMSEES) Laboratory, Faculty of Engineering, Menoufia University, Shebin El-Kom 6131567, Egypt; 5 Department of Mathematics, College of Science and Humanities in Al-Kharj, Prince Sattam bin Abdulaziz University, Al-Kharj 11942, Saudi Arabia; m.abdelgalil@psau.edu.sa; 6 Department of Mathematics, Faculty of Science, Suez Canal University, El-Sheik Zayed, Ismailia 41522, Egypt

**Keywords:** phase change materials (PCM), water fluid, alum sludge, ZnO/Fe_2_O_3_ composite, thermal energy storage, nanofluid

## Abstract

Renewable solar energy storage facilities are attracting scientists’ attention since they can overcome the key issues affecting the shortage of energy. A nanofluid phase change material (PCM) is introduced as a new sort of PCM is settled by suspending small proportions of nanoparticles in melting paraffin. ZnO/α-Fe_2_O_3_ nanocrystals were prepared by a simple co-precipitation route and ultrasonically dispersed in the paraffin to be a nanofluid-PCM. The behaviors of the ZnO/α-Fe_2_O_3_ nanocrystals were verified by X-ray diffraction (XRD) analysis, and the average particle size and the morphology of the nanoparticles were explored by transmission electron microscopy (TEM). For the object of industrial ecology concept, aluminum-based waste derived from water-works plants alum sludge (AS) is dried and augmented with the ZnO/α-Fe_2_O_3_ nanocrystals as a source of multimetals such as aluminum to the composite, and it is named AS-ZnO/α-Fe_2_O_3_. The melting and freezing cycles were checked to evaluate the PCM at different weight proportions of AS-ZnO/α-Fe_2_O_3_ nanocrystals, which confirmed that their presence enhanced the heat transfer rate of paraffin. The nanofluids with AS-ZnO/α-Fe_2_O_3_ nanoparticles revealed good stability in melting paraffin. Additionally, the melting and freezing cycles of nanofluid-PCM (PCM- ZnO/α-Fe_2_O_3_ nanoparticles) were significantly superior upon supplementing ZnO/α-Fe_2_O_3_ nanoparticles. Nanofluid-PCM contained the AS-ZnO/α-Fe_2_O_3_ nanocrystals in the range of 0.25, 0.5, 1.0, and 1.5 wt%. The results showed that 1.0 wt% AS-ZnO/α-Fe_2_O_3_ nanocrystals contained in the nanofluid-PCM could enhance the performance with 93% with a heat gained reached 47 kJ.

## 1. Introduction

The main responsibility of science and technology is signified as the creation of sustainable energy solutions to overcome the substantial use of conventional fossil fuel resources. The globe is suffering from an energy crisis due to the modern lifestyle since the massive gross in population and technological development as well as the energy consumption in the industrial sector [1]. Solar energy is suggested to be a reliable substitution among the accessible renewable energy sources to overcome the energy crisis. However, its availability during the daytime might be diluted and intermittent [2]. Therefore, to satisfy and overcome such inadequacy, energy storage is a must [3]. This technique is based on the capture of sun energy through the daytime to use later, such as thermal energy storage (TES). TES is signified as one of the simple and cost-efficient arrangements and is technically used for energy storage [3]. Numerous ways have been introduced for TES; however, according to the reported literature articles [1,4,5], the proper and widely used is the phase change material (PCM) technology. PCMs suggest numerous solar heat storing capacities in lessening heat losses via the storing period [6]. 

PCM substances appeared in recent decades as the most applicable TES systems and are signified by the way that they are capable of modifying their physical state and proficient in storing TE by discharging their “latent heat” [7]. Such a technique might be attained through melting and solicitation cyclic processes. PCMs might be signified by their presence in at least two structurally different solid phases. These phases could be amorphous phases and one or maybe more in crystalline phase [8,9]. Novel substances, including thermal phase alteration and chemical reactions, are available to be favorable key technologies exhibiting their great TES capacity [10]. The attained TES can be introduced in numerous domestic and industrial applications, i.e., heating, drying, and thermodynamic solar plant industries. Thereby, the research dealing with the TES media applied for using PCM systems is on an upsurge [3,11]. 

PCM-based TES can fall into three categories: (i) sensible heat, (ii) latent heat, and (iii) thermochemical heat storage systems. A sensible heat storage system (SHS) might store energy by temperature difference in the substances in addition to the transformation of the thermal phase altered substances. SHS substances are signified by their superior density, specific heat, and suitable thermal conductivity. Water and rock are the most available sensible heat energy storing examples [11]. However, latent heat thermal energy storage (LHS) is higher than SHS since the enthalpy change through the process of phase change is high. Furthermore, LHS across the SHS showed its superiority since LHS materials possess versatile applications as their excellent heat recovery with small temperature difference signifies high-energy storage density substances [10,11]. PCMs are a class of TES that is contingent on latent heat storage media [8]. Various types of organic (i.e., paraffin-based materials) [12] or inorganic materials (i.e., salt hydrates substances) [8] are applied as PCMs substances for TES facilities. However, thermochemical heat energy storage (TCS) is based on heat storage through chemical bonds based on endothermic or exothermic reactions. However, TCS does not have available commercial applications since it requires phase material development [13,14,15]. Among the various types of TES techniques, latent heat energy storage materials are the most reliable ones since their operating temperature ranges [14]. But, LHS systems are still prerequisites for essential improvement to promote their applications and determine their associated concerns [14,15].

PCMs are based on LHS technology produced by transferring from solid to liquid phase and vice versa on a round cycle [16]. During the phase transition cycles, the energy could be absorbed or released, and the overall energy is stored. Various PCMs are used to overcome sophisticated and expensive systems [12,17,18]. Paraffin-based PCMs are categorized as a good candidate since their wide temperature ranges and superior thermal storage capacity help avoid the problems of supercooling [19,20,21]. However, it is noteworthy to mention that the main shortcoming of the most pristine PCM substances is related to their low thermal conductivity. 

Paraffin material-based PCMs could be used for TES, which might be well insulated for less complicated and inexpensive systems. Paraffin-based substances possess a large amount of latent heat energy, negligible supercooling, and a suitable melting temperature profile [16]. But, the advantage attained by the inclusion of nanoparticle-embedded PCM is resolved in the effective thermophysical properties [17]. For instance, numerous studies have been reported dealing with the enhancement of their critical effectiveness. PCMs are included with various types of nanoparticles [20] to improve their thermal conductivity, including some additives such as carbon nanotubes into paraffin, which is essential for enhancing PCM performances. [22] CuO [23] is embedded in 5 weight% nanoparticles in pure paraffin wax, improving the PCM performance for the melting cycle and enhancing the overall efficiency. ZnO [23] is used as a microencapsulated phase change material integrated into a commercial water tank for cold thermal energy storage improvement. Furthermore, a previous investigation conducted on metal–organic framework (MOF)-based graphene offers numerous merits, including higher thermal conductivity than the pristine PCM [19]. Also, metal–organic PCMs [1] showed superior efficiency in building energy savings. The addition of carbon fiber and carbon nanotubes showed good thermal conductivity [20]. Nano graphite, copper nanowires, titanium carbide, and multiwalled carbon nanotubes also showed an improvement, reaching an 87% increase when just five weight percent is added [21]. However, the application of hybrid metals as a source of nanofluid-PCM to improve their thermal properties is still limited. Also, there is a lack of literature on the use of composite material from waste streams as a source of nanofiller PCM candidates, which is required for further research in such an area. 

Herein, the current phase change thermal energy storage system with spherical capsules was developed. ZnO/α-Fe_2_O_3_ nanocrystals were developed by a simple co-precipitation route. Also, the alum sludge waste derived from water-works plants is dried and mixed with the ZnO/α-Fe_2_O_3_ nanocrystals. Then, the X-ray diffraction (XRD) analysis and the morphology using transmission electron microscopy (TEM) were applied, and the ZnO/α-Fe_2_O_3_ nanocrystals were verified. The feasibility of embedded organic paraffin PCM with ZnO/α-Fe_2_O_3_ nanocrystal to be a ZnO/α-Fe_2_O_3_ nanofluid-PCM for latent heat energy storage was studied. Overall heat gained through the melting/solidification cycles was applied to attain the implementation of the ZnO/α-Fe_2_O_3_ nanocrystal substance base of PCM nanofluid. Hence, the current study presents a novel phase change material system based on the application of waste material as an inexpensive and effective system to attain the benefits of using a nanoparticle composite and a value-added material to improve the PCM system. 

## 2. Experimental Section

### 2.1. Synthesis of ZnO/α-Fe_2_O_3_ Nanoparticles

**Figure 1:**
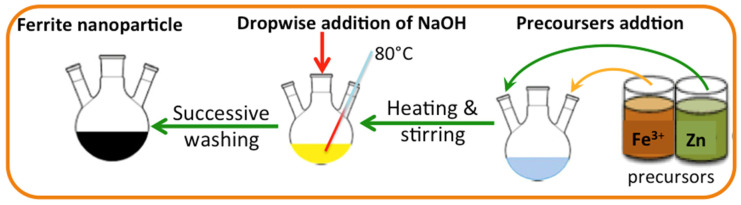
Graphical illustration of the Fe_2_(SO_4_)_3_ and ZnSO_4_ preparation.

Nanosized ZnO/α-Fe_2_O_3_ crystals have been synthesized through the simple co-precipitation route technology as a cost-efficient and straightforward method. The method is applied under a mild temperature range [24], and the essential precursors are added to proceed with the reaction [25]. The analytical grade precursors used during the current co-precipitation route are Fe_2_(SO_4_)_3_ and ZnSO_4_, supplied by Sigma-Aldrich with a purity of 97 and 99%, respectively, and used without any extra purification according to the molar contents of ferrite. The precursor fractions were supplied to the reaction solution media, and thereby, magnetic stirring was applied to attain a homogenous mixture. In a solution of sodium hydroxide, dropwise was added to the mixture to raise the solution pH under heating to reach a black precipitate, which signifies the ferrite formation. Subsequently, the as-synthesized crystals were exposed to successive distilled water washing, and the resultant precipitate is ZnO/α-Fe_2_O_3_ crystals. The graphical presentation of the preparation steps is illustrated in Figure 1. 

In parallel, water-works plant waste is named alum sludge (AS) as a result of using aluminum sulfate as a primary coagulant in the flocculating reservoir. The alum sludge (AS) was collected from a local water-works facility from the underflow channel of the sedimentation tank. Alum sludge is generated by the application of aluminum sulfate as a primary coagulant. Prior to the alum sludge being treated, the excess water is descanted from the sludge by gravity settling and then subjected to open-air drying to reduce the moisture content to only 10%, which is named alum sludge cake. The sludge cake is then subjected to cleaning with distilled water and then oven-dried at 105 °C. The resultant powder is calcinated at 400 °C then ground by a ball mill for one hour. The resultant dried sludge is mixed with the prepared nanoparticles of ZnO/α-Fe_2_O_3_ in a mass proportion of 1:1 weight percent and introduced as an AS-ZnO/α-Fe_2_O_3_ PCM. 

### 2.2. Characterization Study 

The crystal structure of the synthesized ZnO/α-Fe_2_O_3_ nanoparticles was characterized by single-crystal X-ray diffraction (XRD) analysis, which was performed under step-scan mode and conducted via a Bruker–Nonius Kappa CCD diffractometer with CuKα radiation source (λ = 1.5406). The diffractometer works at 40 kV with a scan step time of 0.6 s. Also, the morphology of the synthesized ZnO/α-Fe_2_O_3_ nanoparticles was imaged by SEM micrograph using FE-SEM, Quanta FEG 250. 

### 2.3. Experimental Methodology

A shell-and-tube heat exchanger, STHE, was applied for the melting/solidification cycles of the PCM. Very refined paraffin wax (PW) (95% purity) with a melting point of around 53 °C was chosen as the base PCM. The thermophysical properties of paraffin wax include a latent heat of fusion of 190 kJ/kg, a liquid density of 830 kg/m^3^, and a thermal conductivity of 0.21 kJ/kg °C. The essential amount of PW (15 gm) was melted on a hot plate at 60 °C, followed by AS-ZnO/α-Fe_2_O_3_ nanocrystal addition at a certain weight percent (0.25, 0.5, 1.0, and 1.5 wt%) selected according to the preliminary work. Subsequently, the heterogeneous mixture of PW, as well as AS-ZnO/α-Fe_2_O_3_ nanocrystals, was explored to sonication in an ultrasonic bath and subjected for 30 min sonication at 60 °C (DAIHAN Wisd model WUC-A03H, 40 kHz) to attain the nanofluid-PCM.

**Figure 2:**
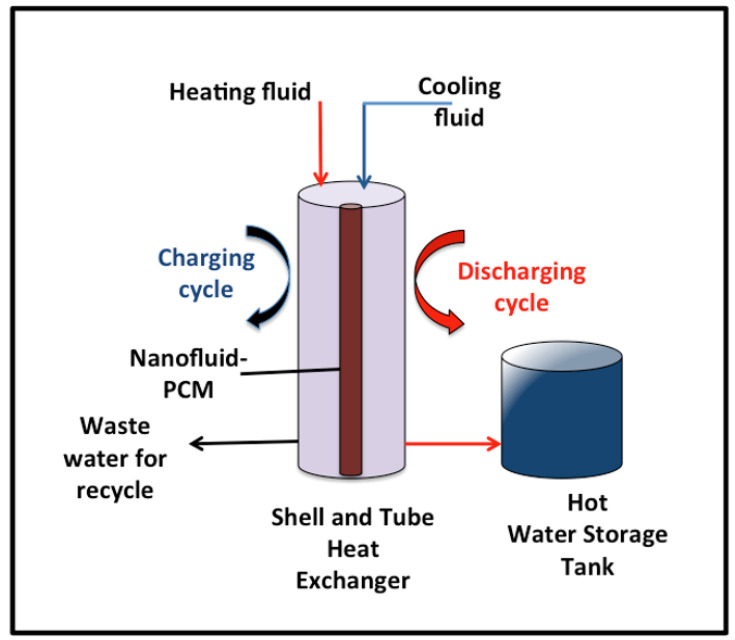
Graphical demonstration of the experimental lab-scale setup.

Initially, 15 gm of PCM is subjected to the shell and tube of the heat exchanger and filled the tube. Water is used as the heat transfer fluid carrier and flows in the shell of the heat exchanger, which supplies the heating and cooling cycles. Heat transfer fluid, water, is exposed to the system at the mass flow rate of 0.0013 kg/s. In order to analyze the system performance, digital thermocouples are mounted to check the melting and solidification temperatures, hot water, and PCM. T-type thermocouples (copper/constantan) are used to investigate the surface temperature of the PCM, as well as the heat transfer fluid. Two thermocouples are monitored in the inlet and outlet heat transfer fluid to record the temperatures and further investigate the heat gained. The thermocouple accuracy is ±0.25 °C accuracy. Thermocouples are subjected to the inlet water and outlet water and inserted in the PCM to monitor the temperatures. All the data are recorded in three replicates, and the average is monitored. After the discharging cycle, the hot water absorbed the heat from the PCM collected in the tank, as shown in Figure 2. The hot water storage tank is well insulated in order to avoid heat losses. The graphical representation of the experimental lab-scale setup is exhibited in Figure 2.

## 3. Results and Discussion

### 3.1. Structural and Morphological Characterization

**Figure 3:**
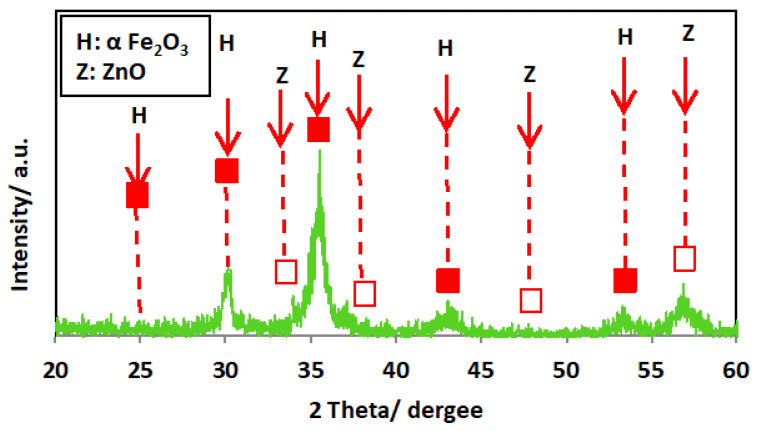
XRD pattern of the as-synthesized ZnO/α-Fe_2_O_3_ nanocrystals.

The XRD diffractogram of the ZnO/α-Fe_2_O_3_ nanocrystals is exhibited in Figure 3. The crystalline phase of the catalyst was investigated, and the XRD patterns showed several diffraction peaks. The spectrum of the XRD diffractogram pattern exposes the formation and presence of the ZnO phases. The presence of the diffraction peak located at 34.4, 36.2, and 47.5 correspond to planes of [002], [101], and [102], and these values correspond to the file (JCPDS Card No.00-005-0664) [26]. Also, the attained XRD pattern verifies that the nanocrystals contain α-Fe_2_O_3_ particles with definite crystalline planes for each peak. The major two peaks are signified at 2θ of 33.1° and 35.6° and are linked to the orientation planes of [104] and [110], respectively, that categorized the presence of α-Fe_2_O_3_. 

**Figure 4:**
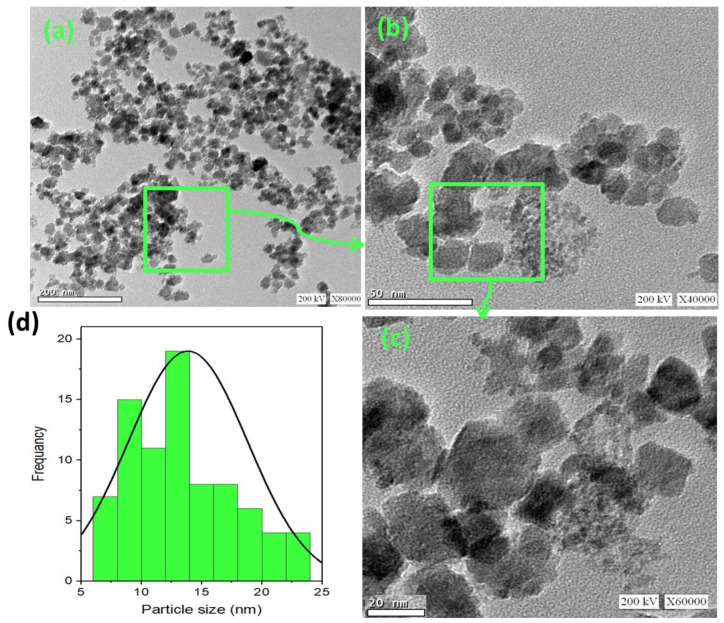
TEM micrographs of (**a**–**c**) as-synthesized ZnO/α-Fe_2_O_3_ nanocrystals at different magnifications with (**d**) particle size histogram.

The morphology of the synthesized ZnO/α-Fe_2_O_3_ nanocrystals was assessed by high-resolution transmission electron microscopy (TEM), and the images are displayed in Figure 4a–c at different magnifications. A TEM micrograph of the ZnO/α-Fe_2_O_3_ nanocrystals revealed a successful construction of the composite ZnO/α-Fe_2_O_3_ nanocrystals in a spherical-like shape. Also, the histogram in Figure 4d exposes that the attained particles are almost spherical in shape with nanoscale size ranges from 6 to 24 nm and the most abundant particle size of about 12.5 nm.

**Figure 5:**
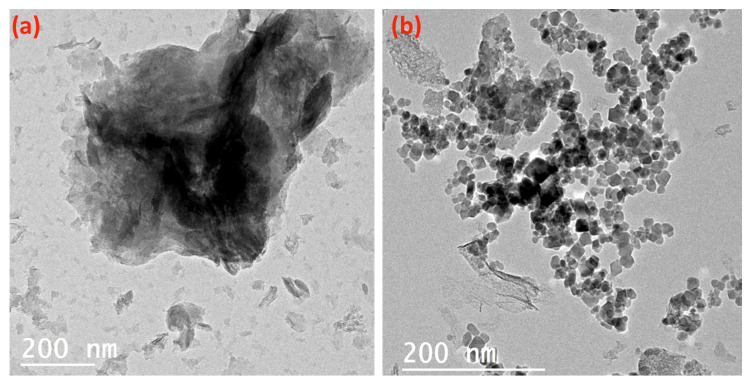
TEM micrograph of the (**a**) alum sludge and (**b**) as-synthesized AS-ZnO/α-Fe_2_O_3_ composite.

Figure 5 displays the TEM images of the alum sludge (Figure 5a) and the AS-ZnO/α-Fe_2_O_3_ composite (Figure 5b). The alum sludge material obviously shows mixed hexagonal-like sheets, as seen in Figure 5a. Furthermore, it is obviously seen from Figure 5b that the composite material showed the alum sludge (AS) showed mixed hexagonal-like particles with uniform distribution attached to its surface with spherical shape particles that signify the ZnO/α-Fe_2_O_3_ nanocrystals. The ZnO/α-Fe_2_O_3_ were well deposited on the surface of alum sludge. However, in some parts of the surface of alum sludge, the ZnO/α-Fe_2_O_3_ nanocrystals became aggregated. Overall, hexagonal-like particles of alum sludge, accompanied by a spherical smaller particle of ZnO/α-Fe_2_O_3_, form the AS-ZnO/α-Fe_2_O_3_ composite material. 

### 3.2. Performance of PCM Analysis

#### 3.2.1. Melting/Solidification Cycles

**Figure 6:**
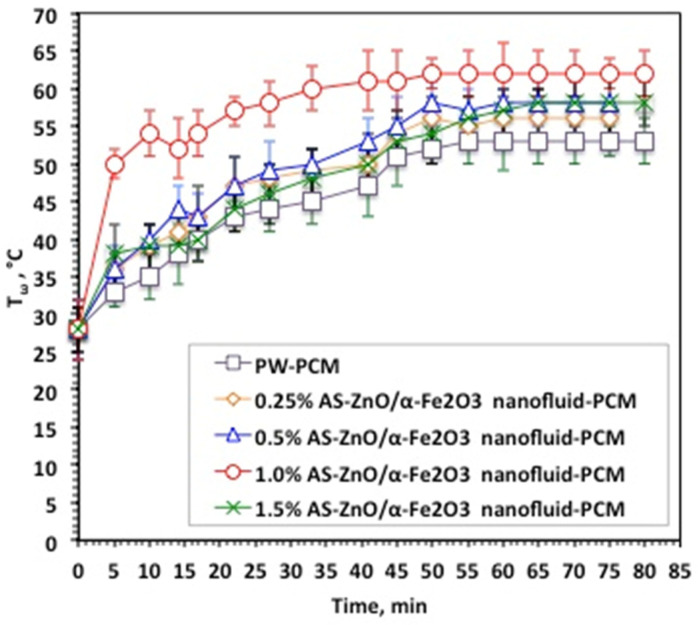
Temperature profile of melting cycle for the solo and AS-ZnO/α-Fe_2_O_3_ nanofluid-PCM systems.

Nanofluid-PCM based on AS-ZnO/α-Fe_2_O_3_ nanocrystals in various systems, as well as the pristine PW-PCM, were subjected to melting (T_ω_) and solidification (T_α_) temperature cycles. The data displayed in Figure 6 exhibited the T_ω_ and T_α_ at different times and for various AS-ZnO/α-Fe_2_O_3_ nanocrystals in nanofluid-PCM systems using 0.25, 0.5, 1.0, and 1.5 wt% of AS-ZnO/α-Fe_2_O_3_ nanocrystals. 

It is distinguished from Figure 6 that AS-ZnO/α-Fe_2_O_3_ nanocrystals in PW result in a range of melting temperatures. An elevation in the temperature is detected by the AS-ZnO/α-Fe_2_O_3_ nanocrystal addition to nanofluid-PCM. The most pronounced system is observable and corresponds to the embedded 1.0% AS-ZnO/α-Fe_2_O_3_ nanofluid-PCM. However, beyond or above this weight percent, the T_ω_ melting starts to reduce. Particularly, this could be illustrated by embedded PCM with the nanoparticles helping in convincing a change in the shape of the heat flow of the thermally changed substance, which thereby adapts the value of the melting temperature of the PCM substance [27,28,29]. Also, AS-ZnO/α-Fe_2_O_3_ addition enhances the latent heat of the PW-based PCM. 

**Figure 7:**
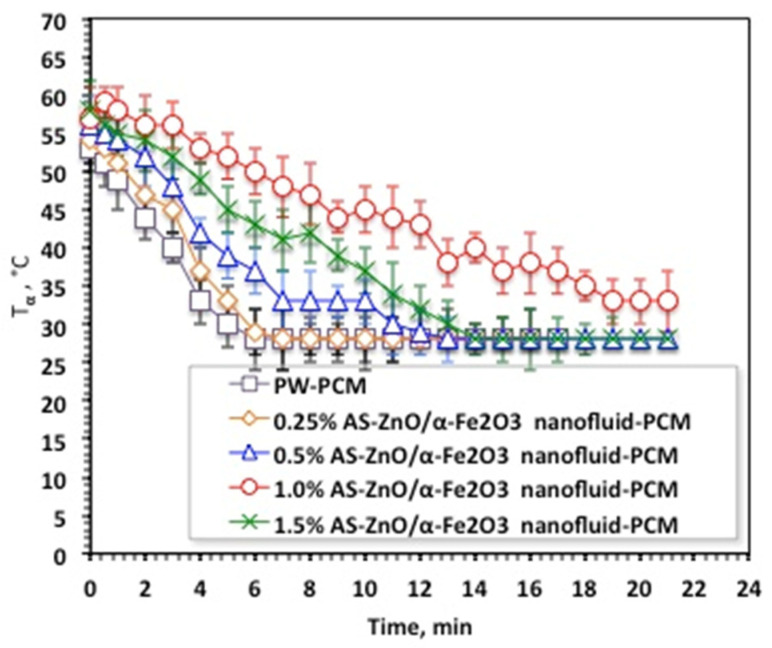
Temperature profile of solidification cycle for the solo and ZnO/α-Fe_2_O_3_ nanofluid-PCM systems.

Solidification cycles of the AS-ZnO/α-Fe_2_O_3_ nanofluid-PCM, as well as the PCM of the pristine PW, are displayed in Figure 7. As the experimental data displayed in Figure 7 shows, the melting temperature with AS-ZnO/α-Fe_2_O_3_ addition into the nanofluid-PCM enhanced the solidification temperature, which thereby is further increased. This could be attributed to AS-ZnO/α-Fe_2_O_3_ addition into PWPCM possessing a higher solidification temperature, which is dependent on the weight percent of the AS-ZnO/α-Fe_2_O_3_ nanoparticle fraction in the nanofluid-PCM. 

The results in Figure 7 noticeably illustrated a relative enhancement of the system by the (1.0%) AS-ZnO/α-Fe_2_O_3_ embedded in the PCM. It is noteworthy to mention that above such amount, T_α_ deduced and thereby the system becomes unfavorable. Such a phenomenon is commonly experimentally observed due to the presence of extra nanoparticles, which might decline the PCM stability, which is unsettled by the agglomeration and sedimentation action. Therefore, choosing the optimal presence of AS-ZnO/α-Fe_2_O_3_ nanoparticles in the nanofluid-PCM is crucial in enhancing the melting/solidification performances. This is in accordance with the previous findings of reported research in the literature [12,30,31,32,33,34]. Notably, it is significant to determine that the AS-ZnO/α-Fe_2_O_3_ nanoparticles could increase the dynamic viscosity of thermally changed substance that results in a diminution in heat transfer rate of the phase change substance [23].

#### 3.2.2. Heat Profile Yield

**Figure 8:**
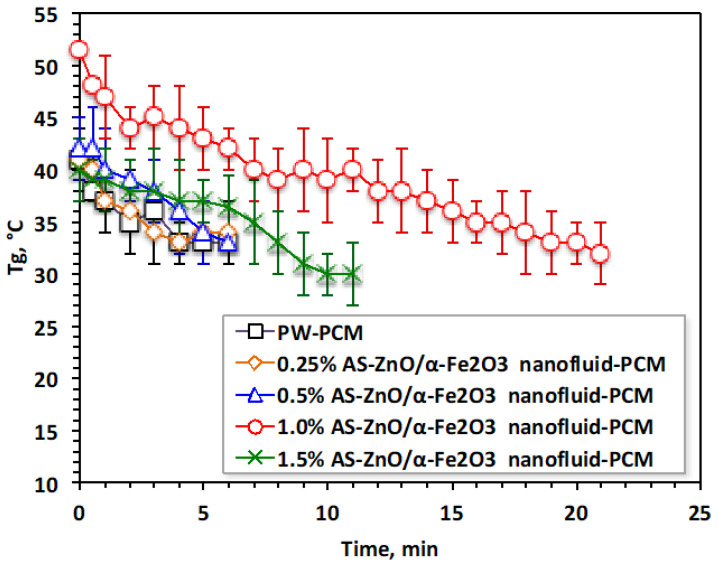
Variation in temperature gained performance for the solo and ZnO/α-Fe_2_O_3_ nanofluid-PCM systems.

Accordingly, choosing the optimal AS-ZnO/α-Fe_2_O_3_ nanofluid-PCM additives results in a heat profile that yields a better range of thermal phase change temperature gained and phase change heat. The gained temperature and heat, $T_{\beta ,}$ and $Q_{\beta }$, respectively, through melting and solidification cycles, are monitored to evaluate the performance of the AS-ZnO/α-Fe_2_O_3_ nanofluid-PCM system. The experimental data illustrated in Figure 8 expose that AS-ZnO/α-Fe_2_O_3_ conjugates in the PW could enhance both the range of temperatures and the amount of acquired heat. Hence, the results are raising the temperature range of the nanofluid-PCM system, which could be applied to a heat storage facility. AS-ZnO/α-Fe_2_O_3_ embedded in PW upsurges the temperature by 15 °C, accordingly increasing the stored-up heat in comparison to the corresponding pristine PW. 

#### 3.2.3. Overall Heat and System Performance

**Figure 9:**
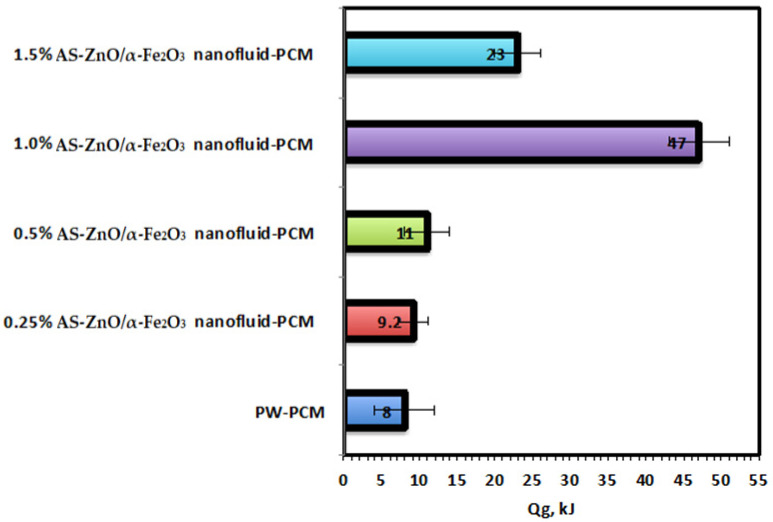
Comparison of overall system heat gained performance for the solo and ZnO/α-Fe_2_O_3_ nanofluid-PCM systems.

Overall, the heat attained by AS-ZnO/α-Fe_2_O_3_ nanofluid-PCM systems, as well as the pristine PCM-based PW system, is calculated and compared. The results displayed in Figure 9 illustrate and compare the heat rate gained by the PCM that is calculated for the whole process for the entire system by Equation (1). The pristine PW and PW conjugates AS-ZnO/α-Fe_2_O_3_ display that the dispersion of AS-ZnO/α-Fe_2_O_3_ increases the overall heat rate achieved from the nanofluid-PCM scheme. According to the experimental data, the useful rate of heat gained is higher for AS-ZnO/α-Fe_2_O_3_ nanofluid-PCM (47 kJ/min) than for the solo PW-PCM system (8 kJ/min). According to this comparison, it is notable that the suggestive greater heat rate gained by the ZnO/α-Fe_2_O_3_ nanofluid-PCM system is linked to the increase in the heat transfer as a result of higher thermal conductivity for the conjugated PW and AS-ZnO/α-Fe_2_O_3_ system [35].

(1) $$Q\upsilon =\dot{w}\;C_{w}\theta_{w}$$

where 

$\dot{w}$: Mass flow rate of HTC (g/s); 

θ: Temperature range between inlet and outlet water entering and leaving the heat exchanger; 

C_w_: Specific heat capacity of heat transfer fluid (4.18 kJ/kg K).

According to the abovementioned data, it is interesting to estimate the overall storing efficiency of the AS-ZnO/α-Fe_2_O_3_ nanofluid-PCM system over the pristine PW solo system. Hence, the overall storing efficiency for all systems is compared and illustrated in Figure 9. Based on the data of the heat gained (Equation (1)) and the heat gained from the PCM substance (Equation (2)), the system efficiency (ƒ) is determined according to Equation (3) [36,37].

(2) $$Q_{PCM}=mC_{p}\left(T_{PCMi}-T_{PCMo}\right)+m\;H$$

where m is the PCM mass (kg), *C_p_* is the specific heat capacity of PCM (kJ/kg K), $T_{PCMi}$ and $T_{PCMo}$ are the inlet and outlet PCM temperature, respectively, from the STHE, and *H* is the latent heat of fusion of PCM (kJ/kg).

(3) $$ƒ=\frac{Q_{\beta }}{Q_{PCM}}\times 100$$

**Figure 10:**
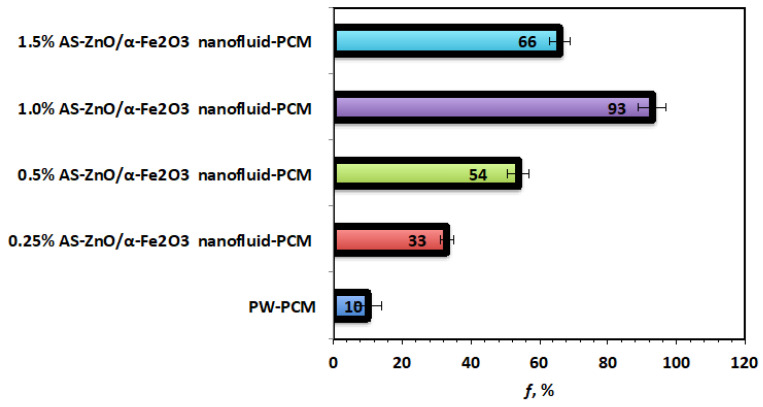
Overall efficiency for the solo and AS-ZnO/α-Fe_2_O_3_ nanofluid-PCM systems.

Figure 10 displays that the efficiency of AS-ZnO/α-Fe_2_O_3_ nanofluid-PCM system and the pristine PW-PCM and the maximum efficiency was observed linked to the (1.0%) AS-ZnO/α-Fe_2_O_3_ nanofluid-PCM. Therefore, the efficiency and heat stored for 1.0% weight AS-ZnO/α-Fe_2_O_3_ nanofluid-PCM is an optimal PCM system. 

Numerous PW-based PCM systems improved through various addition enhancement materials from the literature are concluded and compared with the introduced study. The data presented in Table 1 showed that various systems are enhanced through the various embedded nanofiller substances. As displayed from the data tabulated in Table 1, the upgraded PCM system is achieved from the current work, and a reasonable improvement is attained. 

It is noteworthy to report that, although some other systems showed a more pronounced improvement than the suggested current study, the current investigation is based on the use of only 1% of the nanofiller in comparison to 10% in some cases [38]. The enhancement system proficient from the current study is amongst the greatest values. Applying such inorganic capsulations as the supporting substance accessible thermal behavior greater than the solo paraffin PCM is a promising result.

**Table 1:** Comparison of nanoparticle-enhanced PW-PCM systems investigated through various studies *.

Nanomaterial	Addition Weight (%)	Key Results	Application	Ref.
AS-ZnO/α-Fe_2_O_3_	1.0%	Efficiency increased 93%	Heating	Current investigation
Al_2_O_3_	2.0%	Temperature enhanced with 1.5 °C	Heating	[39]
SiO_2_	NA	Efficiency increased 9%	Heating	[40]
TiO_2_	1.0%	Reduce in PCM heat by 0.5%	NA	[12]
CuO	0.02%	Increase in thermal conductivity	Heating	[41]
ZnO	3.0%	Thermal conductivity increased	Heating	[42]
ZnO/SiO_2_	3.0%	Efficiency increased 19%	Heating	[43]
ZSM-12	NA	Temperature enhanced with 21 °C	Heating	[44]
Carbon	1.0%	Thermal conductivity increased 49%	Heating	[45]
Carbon	10%	Thermal conductivity increased 31%	Photovoltaic cells	[38]
Carbon nanotube	5%	Thermal conductivity increased 87%	Heating	[21]
Ag	10%	Thermal conductivity increased	Heating	[46]
carbon nanofiber	10%	Thermal conductivity enhancement 31%	photovoltaic cells	[47]
MgO	1%	Thermal conductivity enhancement 17%	Supercooling	[48]
nanographene		Thermal conductivity enhancement 10%	Water heating	[49,50]
PCM containing mortar		A reduction of almost 10 °C	Ventlation	[51]
PCM in the gypsum		Thermal dynamic characteristics improved 45%	Thermal inertia of buildings	[52]
Double layer PCM		Energy saving 38%	Building’s energy efficiency and thermal comfort	[53]

* NA: not available.

## 4. Conclusions

Experimental work was explored, and data were used to assess the thermophysical characteristics through melting and solidification cycles for both solo pristine paraffin wax and AS-ZnO/α-Fe_2_O_3_ nanofluid-PCM systems. A mixture of 0.25, 0.5, 1.0, and 1.5% by weight of AS-ZnO/α-Fe_2_O_3_ nanoparticles was embedded into the paraffin wax as the base material. The nanofluid-PCM systems displayed a superior thermal behavior displayed in terms of melting/solidification cycles. The addition of nanoparticles enhanced thermal heat storing capacity compared to the pristine paraffin wax PCM system. For 1.0 wt% nanoparticles of the AS-ZnO/α-Fe_2_O_3_ nanofluid-PCM system, the heat storing capacity showed the highest among the proposed systems. By considering such data, it can be concluded that the composite thermally phase change substance with 1.0 wt% with 1.0 wt% ZnO/α-Fe_2_O_3_ nanoparticles could be a potential candidate to store energy. Thus, further future work is required to harvest solar energy using flat plat collectors using such a proposed system for building heating applications due to its superior thermal consistency.

## Data Availability

Data are available upon request.

## Nomenclature

PCMs	Phase change material
TES	thermal energy storage
XRD	X-ray diffraction analysis
TEM	transmission electron microscopy
STHE	Shell-and-tube heat exchanger
LHS	Latent heat thermal storing
SHS	Sensible heat storing
TCS	thermochemical heat energy storage
PW	Paraffin wax
T_ω_	Melting temperatures, °C
T_α_	Solidification temperatures, °C
T_β_	Gained temperature after storing, °C
$\dot{w}$	Mass flow rate of heat transfer fluid, g/s
θ	Temperature range between inlet and outlet water
C_w_	Specific heat capacity of heat transfer fluid, kJ/kg K
ƒ	Overall system efficiency, %
